# Decreased methylation in the *SNAI2* and *ADAM23* genes associated with de-differentiation and haematogenous dissemination in breast cancers

**DOI:** 10.1186/s12885-018-4783-x

**Published:** 2018-09-06

**Authors:** Lenka Kalinkova, Iveta Zmetakova, Bozena Smolkova, Gabriel Minarik, Tatiana Sedlackova, Viera Horvathova Kajabova, Zuzana Cierna, Michal Mego, Ivana Fridrichova

**Affiliations:** 1 Department of Genetics, Cancer Research Institute, Biomedical Research Center, Slovak Academy of Sciences, v.v.i., Dubravska cesta 9, 845 05 Bratislava, Slovak Republic; 2 Institute of Molecular Biomedicine, Faculty of Medicine, Comenius University, Sasinkova 4, 811 08 Bratislava, Slovak Republic; 3 Institute of Pathological Anatomy, Faculty of Medicine, Comenius University, University Hospital, Sasinkova 4, 811 08 Bratislava, Slovak Republic; 4 2nd Department of Oncology, Faculty of Medicine, Comenius University, National Cancer Institute, Klenova 1, 83310 Bratislava, Slovak Republic

**Keywords:** *SNAI2* methylation, *ADAM23* methylation, Breast cancer, Cancer cell de-differentiation, Haematogenous dissemination

## Abstract

**Background:**

In breast cancer (BC), deregulation of DNA methylation leads to aberrant expressions and functions of key regulatory genes. In our study, we investigated the relationship between the methylation profiles of genes associated with cancer invasivity and clinico-pathological parameters. In detail, we studied differences in the methylation levels between BC patients with haematogenous and lymphogenous cancer dissemination.

**Methods:**

We analysed samples of primary tumours (PTs), lymph node metastases (LNMs) and peripheral blood cells (PBCs) from 59 patients with sporadic disseminated BC. Evaluation of the DNA methylation levels of six genes related to invasivity, *ADAM23*, *uPA*, *CXCL12*, *TWIST1*, *SNAI1* and *SNAI2*, was performed by pyrosequencing.

**Results:**

Among the cancer-specific methylated genes, we found lower methylation levels of the *SNAI2* gene in histologic grade 3 tumours (OR = 0.61; 95% CI, 0.39–0.97; *P* = 0.038) than in fully or moderately differentiated cancers. We also evaluated the methylation profiles in patients with different cancer cell dissemination statuses (positivity for circulating tumour cells (CTCs) and/or LNMs). We detected the significant association between reduced DNA methylation of *ADAM23* in PTs and presence of CTCs in the peripheral blood of patients (OR = 0.45; 95% CI, 0.23–0.90; *P* = 0.023).

**Conclusion:**

The relationships between the decreased methylation levels of the *SNAI2* and *ADAM23* genes and cancer de-differentiation and haematogenous dissemination, respectively, indicate novel functions of those genes in the invasive processes. After experimental validation of the association between the lower values of *SNAI2* and *ADAM23* methylation and clinical features of aggressive BCs, these methylation profiles could improve the management of metastatic disease.

**Electronic supplementary material:**

The online version of this article (10.1186/s12885-018-4783-x) contains supplementary material, which is available to authorized users.

## Background

Breast cancer (BC) is one of the most common tumours occurring in women worldwide, with high mortality rates [1]. In recent population study, it was observed that of 13,785 BC patients undergoing adjuvant chemotherapy, 24.4% developed metastatic disease, with median survival of 20 months. In contrast to previous studies, no general improvement of survival in metastatic recurrent BC patients after chemotherapy has been evidenced over the last three decades [2]. Therefore, to assist in the development of effective diagnostic and therapeutic strategies for BC, the identification of more precise biomarkers is necessary.

It is generally accepted that during the initiation and progression of cancer, deregulation of epigenetic processes, including DNA methylation, occurs and results in aberrant expression and function of a number of key regulatory genes [3, 4]. Changes in DNA methylation in primary tumours (PTs) compared with normal breast tissues have been partially identified by numerous researchers; however, whole-genome bisulfite sequencing technology is now available, which allows the comprehensive analysis of normal and BC methylomes [5].

The metastatic cascade involves multiple steps enabling the detachment of cancer cells from PTs, their intravasation to the circulatory system, their avoidance of the immune reaction and their migration through the circulation. Some tumour cells can extravasate from vessels and invade distant organs. In a new microenvironment, tumour cells can persist in a dormant stage or form micrometastases, which can later develop into macrometastases [6–9].

The critical processes underlying the spread of cancer cells include the degradation of the basement membrane (BM) and the extracellular matrix (ECM). The BM is formed by dense interconnected sheets of ECM proteins, and the loss of tissue architecture and the disruption of structural boundaries between the epithelium and the stroma are the typical features of advanced stages of malignancy [10]. ADAMs (a disintegrin and metalloproteases) have been defined as transmembrane proteins with protease and adhesion functions [11]. ADAM23 belongs to the ADAM protein family although it does not have metalloprotease activity; however, its disintegrin-like domain facilitates its interaction with integrins, resulting in integrin-dependent cell adhesion [12–14]. Cancer cells not expressing *ADAM23* have higher migration capacities in cell line models [15]. Moreover, promoter hypermethylation of the *ADAM23* gene in breast PTs is significantly associated with more advanced grade, higher proliferation of cancer cells, poor prognosis and lower survival rates [15–17].

Cancer cells contribute to the degradation of the ECM by secreting various proteolytic enzymes, such as urokinase plasminogen activator (uPA). uPA is a serine protease that contributes to the conversion of inactive plasminogen to plasmin and that, in cooperation with the uPA receptor (uPAR), degrades the fundamental ECM components, such as laminin and fibronectin [18]. Several in vitro and in vivo studies have documented the essential role of uPA in tumour growth, the detachment of cancer cells from PT and haematogenous dissemination [19, 20]. Moreover, the prognostic impact of uPA and plasminogen activator inhibitor-1 (PAI-1) expression levels has been identified predominantly in patients with HER2+ tumours [21]. Experimental studies have documented that promoter methylation is the dominant mechanism of *uPA* silencing in BC progression [22, 23]. Previously, the complete demethylation of the *uPA* gene and expression of *uPA* mRNA was found only in highly invasive oestrogen receptor-negative BC cell lines; in normal and low invasivity lines, *uPA* hypermethylation with reduced mRNA expression was observed [24]. In BC patients, *uPA* hypomethylation has been associated with metastatic disease, but the *uPA* hypermethylation in non-selected BC was evidenced using the more complex methylation analyses of multiple CpG islands [24, 25].

Invasion by cancer cells occurs at the interface between tumour and host tissue, and the intensive exchange of many enzymes, chemokines and cytokines contributes to the destruction of the BM, the local modulation of the ECM and the stimulation of cancer cell migration [26]. Chemokine CXC motif ligand 12 (CXCL12), which is produced at high levels by cells of the tumour microenvironment and target tissues including lymph nodes, interacts with its signal receptor, CXCR4, expressed on the surface of cancer cells. Using the chemotactic gradient of CXCL12-CXCR4, CXCL12 participates in the regulation of the metastatic process [27, 28]. Both encoding genes, *CXCL12* and *CXCR4*, are epigenetically regulated during the modulation of metastatic potential in BC. In patients with histologically advanced disease and worse survival, *CXCL12* methylation and *CXCR4* overexpression without methylation have been found [29–31]. In our previous study we documented the relationship between *CXCL12* hypermethylation and increased risk for the development of LNMs in BC patients [17].

For the infiltration of epithelial cancer cells into circulation, it is necessary for them to shift into a more mesenchymal phenotype through the epithelial-mesenchymal transition (EMT). In normal physiological development, EMT is active in embryonal and organ development. In epithelial tumours, EMT contributes to the loss of cancer cell polarity, cell-cell contacts and cell-BM interactions. Owing to these mesenchymal characteristics, cancer cells obtain the capacity to migrate and invade, and they became be more resistant to apoptosis [32]. The EMT process is regulated by three main families of transcription factors (TFs), Zeb, Snail and Twist. Of these TFs, ZEB1 and ZEB2 (zinc finger E-box binding homeobox 1/2), SNAI1 and SNAI2 (snail family zinc finger 1/2) and TWIST1 and TWIST2 (basic helix-loop-helix transcription factor 1/2) are known as direct regulators of the adhesion molecule E-cadherin [33]. Using immunohistochemical analyses, changes in Twist, Snail and Slug expressions have been observed in BC. In patients with metastatic disease, increasing levels of TWIST protein are associated with cancer-caused death, and higher levels of SLUG (SNAI2) protein are present in cases with higher tumour grades [34]. More prevalent *TWIST1* hypermethylation has been observed in BC compared to normal breast tissue; however, no direct evidence of the epigenetic regulation of *TWIST1* mRNA or the relevant protein expression has been found [35–37]. Higher *TWIST1* methylation levels have been evidenced in non-triple negative (TN) BC than in TN BC and in ductal compared to lobular invasive carcinomas [38, 39]. The dynamic changes in DNA methylation in genes encoding other two EMT-TFs, *SNAI1* and *SNAI2,* have been observed in a EMT/MET cell-line model, where increased methylation was associated with decreased transcription in both genes [40]. In a previous in vitro study, regardless of the significantly higher expression levels of both *SNAI2* and Z*EB2* genes in metastatic BC cell lines, only moderate DNA methylation differences were found in *SNAI2* between highly and poorly metastatic lines, which was in contrast to the *ZEB2* gene, which was markedly hypomethylated in highly metastatic cell lines [41]. These results indicate more complex interactions in the epigenetic regulation of cancer invasivity including EMT.

Cancer cells disseminate from PTs either via blood vessels after intensive neo-vascularisation (haematogenous spread) or via the lymphatic system, named lymphogenous spread, which occurs after neo-lymphangiogenesis [42, 43]. In cancers, the active growth of new blood vessels from pre-existing ones is triggered by tumour cells expressing vascular endothelial growth factor A (VEGFA) in the hypoxic microenvironment of tumour tissue. New angiogenic blood vessels are abnormal, with a lack of pericytes and BM connection and with some fenestrations, allowing intravasation and the haematogenous spread of cancer cells [44]. In BC, it has been found that endothelial cells produce soluble factors, such as hepatocyte growth factor (HGF), which contribute to EMT [45]. Several studies have shown that in numerous cancer types including BC, the neo-vascularization associated with the presence of circulating tumour cells (CTCs) in blood vessels and the higher number of CTCs are correlated with poor prognosis and decreased overall survival [46–49]. On the other hand, tumour cells can express and secrete several lymphangiogenic factors, including VEGFC/D, to promote the formation of new lymphatic vessels in the tumour stroma [50]. From the lymphatic vasculature, cancer cells can enter the blood circulation via the thoracic duct or form metastases in lymph nodes. Moreover, cancer cell motility and spread are affected by the density of blood and lymphatic vessels, the interstitial blood pressure, tumour hypoxia, regional lymph node metastases (LMNs) and the recently discovered primo vascular system [42]. The preferred dissemination route of cancer cells depends on cancer types and microenvironmental conditions, but it has already been reported that carcinomas, such as BC, and melanomas more often spread through the lymphatic vessels and develop LNMs, in comparison to soft tissue sarcomas, which predominantly result in systematic metastasis [51, 52].

In our previous study of methylation profiles in 206 BC patients we showed that the risk for LNMs development and higher proliferation of cancer cells was increased by hypermethylation of *CXCL12* and *ADAM23* genes, respectively [17]. For present study, we selected 59 BC patients with haematogenous and/or lymphogenous cancer dissemination to investigate more deeply the relationship between the DNA methylation patterns and the clinico-pathological parameters of more aggressive BC. In addition to *CXCL12*, *ADAM23* and *uPA* genes contributing to the inhibition of cell adhesion, BM degradation and ECM remodelling, *TWIST1, SNAI1* and *SNAI2* genes, the protein products of which are known as key inducers of EMT, were involved. The changes in methylation levels of patients with haematogenous and/or lymphogenous dissemination of BC could help to identify potential biomarkers for metastatic potential of BC alone and also for different routes of metastatic progression.

## Methods

### Patients and samples

In the present study we analysed PT tissues and LNM samples, both isolated from formalin fixed paraffin-embedded tissues (FFPE) and peripheral blood cells (PBCs) from 59 selected patients with disseminated BC. Their tumours were considered as sporadic, because these patients did not fulfil criteria for hereditary BC testing. PBC samples from 53 healthy women and 10 normal mammary gland samples from mammoplasties were used as controls. PT and LNM specimens were collected at the National Cancer Institute in Bratislava, Slovakia between 2012 and 2014, and the control samples were collected in previous studies. This study was approved by the Institutional Review Board of the National Cancer Institute of Slovakia, and written informed consent was obtained from all patients and controls. The clinical and histopathological characteristics were obtained from patient records, and the tumours were defined according to TNM classification. The clinico-histopathological data of evaluated patients are summarized in Table 1 [53]. No patient underwent preoperative radiotherapy or chemotherapy before sample collection. The age of the women with BC ranged from 37 to 79 years (mean 60.19 ± 10.38 years). The PBC samples were obtained from control women aged 40 to 85 years (mean 58.17 ± 10.50 years). The age of the normal breast tissue donors ranged from 31 to 56 years (mean 46.90 ± 9.06 years). No control persons had signs or symptoms of cancer or other serious diseases.

**Table 1:** Clinical characteristics

Variables		*n*	%
All		59	100.0
Age	≤50	13	22.0
	> 50	46	78.0
Histology	DIC	49	83.1
	Others	10	16.9
Tumour size (mm)	≤20	34	57.6
	> 20	25	42.4
LNM status^a^	0	17	28.8
	≥1	42	71.2
TNM classification	I	14	23.7
	II	22	37.3
	III	23	39.0
Grade	1 and 2	36	63.2
	3	21	36.8
Hormone receptor status^b^	Negative	8	13.6
	Positive	51	86.4
HER2 status^c^	Normal	16	27.1
	Amplified	43	72.9
Ki-67 proliferative index^d^	Low	35	59.3
	High	24	40.7
Tumour multifocality	Negative	51	87.9
	Positive	7	12.1
CTC any^e^	Negative	30	50.8
	Positive	29	49.2
CTC epithelial	Negative	45	76.3
	Positive^f^	14	23.7
CTC mesenchymal	Negative	40	67.8
	Positive^g^	19	32.2
CTC^e^ and LNM status	Negat. / Posit.	29	50.0
	Posit. / Negat.	16	27.6
	Posit. / Posit.	13	22.4

*Abbreviations: DIC* ductal invasive carcinoma, *LNM* lymph node metastasis, *HER2* human epidermal growth factor receptor 2, *CTC* circulating tumour cell

^a^LNM status was categorized according to the number of metastatic LNs

^b^Negative for both (oestrogen receptor and progesterone receptor) or positive for either with cut-off 10%

^c^HER2 status was determined immunohistochemically according to ASCO guidelines [53]

^d^Cut-off 20%

^e^CTCs were detected through the quantification of EMT-inducing transcription factor gene transcripts

^f^Out of them 4 BC patients had simultaneously mesenchymal CTC

^g^Out of them 4 BC patients had simultaneously epithelial CTC

### DNA extraction and sodium bisulfite DNA modification

The representative samples of PTs (minimally 80% of cancer cells), LNMs and healthy tissues from mammary glands were selected from FFPE blocks. Tissue samples identified under light microscope were marked and 3 mm diameter cores of tissue were removed from donor blocks into the recipient master block using a tissue microarray method, as described previously [54]. DNA was extracted by the MagneSil Genomic Fixed Tissue System (Promega, Madison, Wisconsin, USA). DNA from PBCs was isolated using the FlexiGene DNA Kit (Qiagen, Hilden, Germany). All extraction methods were performed according to the manufacturer’s instructions. A NanoDrop 1000 spectrophotometer (Thermo Fisher Scientific, Bremen, Germany), was used to measure DNA concentration. DNA bisulfite modification was performed by the EpiTect Bisulfite Kit (Qiagen, Hilden, Germany) and the CpGenome DNA Modification Kit (Chemicon, Temecula, California, USA) for FFPEs (2 μg of isolated DNA) and PBC samples (1 μg of isolated DNA), respectively. The samples of modified DNA were divided into aliquots and stored at − 18 °C. The principle of DNA bisulfite modification is the conversion of unmethylated cytosines to uracils, while methylated cytosines remain unchanged.

### PCR and methylation analyses

For the evaluation of the DNA methylation profiles in the six selected cancer-associated genes (*ADAM23*, *uPA, CXCL12, TWIST1*, *SNAI1* and *SNAI2*), the quantitative pyrosequencing method was used. Between 3 and 8 CpGs were analysed in each gene in the CpG islands of the promoter regions flanking the transcription start site. The primers for PCR and the pyrosequencing reactions were designed by PyroMark Assay Design software 2.0 (Qiagen, Hilden, Germany). The primers are shown in Table 2. All the designed assays were validated according to the manufacturer’s instructions. The PCR reactions were performed by the PyroMark PCR Kit (Qiagen, Hilden, Germany) following manufacturer’s instructions. The concentrations of the PCR primers were 0.32 μM for *uPA*, *CXCL12, TWIST1*, *SNAI1*, and *SNAI2* and 0.40 μM for *ADAM23,* with the annealing conditions of 56 °C for 30 s except for the *ADAM23* gene, for which 52 °C for 30 s was used. For pyrosequencing, the PyroGold Reagent Kit (Qiagen, Hilden, Germany) and PyroMark Q24 System were used, and the results were evaluated by PyroMark Q24 2.0.6 software (Qiagen, Hilden, Germany). Methylation levels were defined as the percentage of average methylation in all CpG sites in each individual gene.

**Table 2:** Primers for PCR and pyrosequencing

Gene	Orienta-tion	Sequence (5′-3′) of PCR primer	PCR product (bp)	Sequence (5′-3′) of pyrosequencing primer	Numb. of CpGs
*ADAM23* ^a^	Forward Reverse	[Biotin]GCGTCGTTTTAGTATTTTTAGGTT TCCCCAACCACTACTCCCT	89	ACTACTCCCTCCCCC	8
*uPA*	Forward Reverse	TAGGTGTATGGGAGGAAGTA [Biotin]CTCCCTCCCCTATCTTACA	165	GTTTTTTTTAAATTTTTGTGAG	7
*CXCL12* ^b^	Forward Reverse	TAGTGGGGTTTTGTTATAGGGATA [Biotin]ACCTTTAACCTTCTCAAACTC	121	GGGTTTTGTTATAGGGATAAT	7
*TWIST1*	Forward Reverse	GAAGGGGAGGGAAGGGG [Biotin]TAACAATTCCTCCTCCCA	97	GGGAGGGAAGGGGGAG	3
*SNAI1*	Forward Reverse	GTATTTGTTAGGGGAGTGGT [Biotin]ACCACCCCCCTTTATCAC	72	GTTAGGGGAGTGGTTT	6
*SNAI2*	Forward Reverse	[Biotin]GTTGGTTTGGTGTGGTGTAG CCCTACCCCCCTAACTTCCAAATATAAT	100	CCTAACTTCCAAATATAATACAAC	5

^a,b^Set of PCR primers and pyrosequencing primers were previously published by Zmetakova and colleagues [54]

### CTCs detection in peripheral blood

CTCs were detected in 5 mL of peripheral blood depleted of CD45-positive (CD45+) cells for CTC enrichment using a quantitative real-time polymerase chain reaction (qRT-PCR) assay, as described previously [55, 56]. Patient samples with higher levels of *KRT19* gene transcripts than those of healthy donors were scored as epithelial CTC-positive, while patient samples with higher mRNA levels of EMT-transcription factors (*TWIST1*, *SNAI1, SLUG* (*SNAI2*) and *ZEB1*) than those of healthy donors were scored as mesenchymal CTC-positive. The expression of at least one of the markers (either epithelial or mesenchymal) at levels above the defined cut off was sufficient to define a sample as CTC-positive.

The highest expression levels of the *KRT19* and EMT-inducing TF gene transcripts relative to that of *GAPDH* were 3.4 × 10^− 3^ (median 2.8 × 10^− 6^, range: 0–3.4 × 10^− 3^) for *KRT19*, 7.5 × 10^− 4^ (median 0, range: 0–7.5 × 10^− 4^) for *TWIST1*, 3.8 × 10^− 2^ (median 3.1 × 10^− 3^, range: 5.0 × 10^− 4^ - 3.8 × 10^− 2^) for *SNAIL1* and 1.7 × 10^− 1^ (median 1.4 × 10^− 2^, range: 2.2 × 10^− 3^ - 1.7 × 10^− 1^) for *ZEB1*, while *SLUG* transcripts were not detected in any of the samples from healthy donor. These highest expression values in healthy donors were used as “cutoff” to determine CTCs positivity [57].

### Statistical analyses

SPSS statistics 15.0 software was used for the statistical analyses of the data. The normality of distribution was tested by the Shapiro-Wilk test. Normally distributed samples were tested by Student’s t-test or analysis of variance (ANOVA) with Bonferroni’s corrections. Nonparametric Mann-Whitney U or Kruskal-Wallis H tests were used for non-normally distributed data. According to the normality of data, Pearson’s or Spearman’s correlations were used. Univariate analyses were performed for categorical variables using χ2 or Fisher’s exact test. A statistical two-tailed significance was regarded as *P* value < 0.05. A logistic regression adjusted for age was used to determine the effect of independent categorical variables and DNA methylation on the clinical status and tumour dissemination.

## Results

### DNA methylation in various sample types in BC patients and controls

In this study, we evaluated specific DNA methylation levels by the quantitative pyrosequencing method in three types of samples isolated from 59 BC patients, namely, in PT and LNM tissues and PBCs. From the six analysed genes, higher DNA methylation levels were found in PTs for *ADAM23*, *uPA*, *CXCL12* and *TWIST1* genes, with values of 9.98%, 14.08%, 13.41% and 21.88%, respectively. Similar methylation levels of these four genes were found in LMN samples, and a positive correlation between PT and LNM tissues was observed (*r* = 0.598, *P* = < 0.001; *r* = 0.498, *P* = 0.011; *r* = 0.380, *P* = 0.013; *r* = 0.428, *P* = 0.026; respectively). In BC patients, increased methylation levels were detected in PT and LNM samples compared with PBCs for all studied genes. Methylation results in different types of samples are summarized in Table 3.

**Table 3:** The mean DNA methylation levels in healthy women and breast cancer patients in different types of samples

	DNA methylation levels – controls Mean ± std. deviation (in %)		DNA methylation levels – breast cancer patients Mean ± std. deviation (in %)		
Genes	Peripheral blood cells	Mammary glands^a^	Peripheral blood cells	Primary tumour	Lymph node metastasis
*ADAM23*	2.13 ± 0.39	4.89 ± 2.32	2.50 ± 0.64	9.98 ± 10.32	9.94 ± 10.89
*uPA*	1.29 ± 0.64	5.00 ± 3.80	2.20 ± 0.83	14.08 ± 16.88	9.45 ± 12.49
*CXCL12*	2.36 ± 0.52	4.00 ± 1.94	2.78 ± 1.14	13.41 ± 13.32	11.35 ± 9.81
*TWIST1*	4.44 ± 1.15	8.20 ± 6.29	4.15 ± 1.42	21.88 ± 16.68	15.23 ± 12.90
*SNAI1*	1.46 ± 0.58	3.80 ± 2.04	2.17 ± 1.01	6.04 ± 6.71	4.60 ± 2.04
*SNAI2*	2.37 ± 0.63	3.30 ± 1.34	3.46 ± 0.82	6.37 ± 4.49	5.44 ± 5.79

^a^Donors of normal breast tissues were younger than breast cancer patients

Furthermore, in univariate analysis, we found significantly different methylation levels in PBC samples between BC patients and controls for *ADAM23*, *uPA, SNAI1 and SNAI2*, with *P* values ≤0.001 for all. Similarly, significant differences in DNA methylation between PT and normal breast tissues were observed for *CXCL12, TWIST1* and *SNAI2* genes (*P* = 0.001, *P* = 0.006 and *P* = 0.009, respectively). However, these results cannot be correctly interpreted for ageing processes interference with DNA methylation in patient’s tumour tissue samples.

### DNA methylation and clinico-histopathological characteristics

**Fig. 1:**
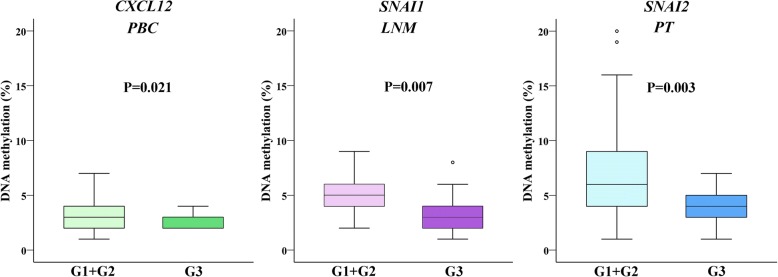
DNA methylation levels in patients with different histologic grade in three types of samples for *CXCL12, SNAI1* and *SNAI2* genes. The length of the boxes is the interquartile range (IQR) that represents values between the 75th and 25th percentiles. The circle (○) labelled outliers (values more than 1.5 IQRs but less than 3 IQRs from the end of the box). Values more than three IQRs, extremes, are depicted by asterisk (*).The horizontal line represents median. Significance level is regarded as *P* < 0.05. Abbreviations: PBC, peripheral blood cells; PT, primary tumour; LNM, lymph node metastases; G, histologic grade

In the group of 59 BC patients, the relationships between the DNA methylation levels of all six evaluated genes and the clinico-histopathological features such as tumour histology, tumour size, LNM status, TNM, grade (G), hormonal receptor (HR) and HER2 status, Ki-67 proliferative index, tumour multifocality and CTC presence were analysed in PT, LNM and PBC samples. We identified significant differences in methylation levels between patients with G1 and G2 BC compared to those with G3 BC. Compared to patients with G3 BC, patients with lower grades presented higher methylation levels in three genes: *CXCL12* in PBCs, *SNAI1* in LNMs and *SNAI2* in PT samples. The mean methylation levels in G1 and G2 compared to G3 for those three genes were 3.06% vs. 2.35%, 5.39% vs. 3.42%, and 7.38% vs. 4.00% with *P* values of 0.021, 0.007, and 0.003, respectively (Fig. 1). Methylation results for all six genes in patients with different histological grade are summarized in Additional file 1.

The relationship between grade and *SNAI2* methylation in tumour tissue was confirmed by multivariate logistic regression analysis. The influence of the methylation of EMT-associated genes (*TWIST1*, *SNAI1* and *SNAI2*) and the clinico-histopathological features of invasive BC, namely, age, HR and HER2 status, tumour histology and Ki-67 proliferative index on tumour grade were assessed in a multivariate model. The methylation of the *SNAI2* gene negatively associated with higher tumour grade (grade 3) (OR = 0.61; 95% CI, 0.39–0.97; *P* = 0.038) and higher grade positively associated with the high Ki-67 proliferative index (OR = 17.34; 95% CI, 2.89–103.88; *P* = 0.002) (Table 4).

**Table 4:** The risk estimation of analysed variables and clinical status for histological grade 3 in breast cancer patients (logistic regression adjusted for age)

	Variables	*P* value	OR	95% CI
Grade 3	↑ Methylation of *SNAI2* in PT	0.038	0.61	0.39–0.97
	Ki-67 proliferation index > 20%	0.002	17.34	2.89–103.88

−2 Log likelihood = 31.62; *R*^2^(Cox and Snell) = 0.43; *R*^2^(Nagelkerke) = 0.60

*Abbreviations*: *PT* primary tumour, *OR* odds ratio, *CI* confidence interval, ↑ continuous variable. Age and DNA methylation of EMT related genes, hormone receptor and HER2 status, histological type and Ki-67 proliferative index were analysed as independent variables, significant results are shown in the table

### DNA methylation and dissemination of tumour cells

In the present study, we investigated the differences in methylation profiles between patients with haematogenous dissemination of cancer cells, indicated by the presence of CTCs in peripheral blood, and/or lymphogenous spread, represented by the presence of LNMs.

**Fig. 2:**
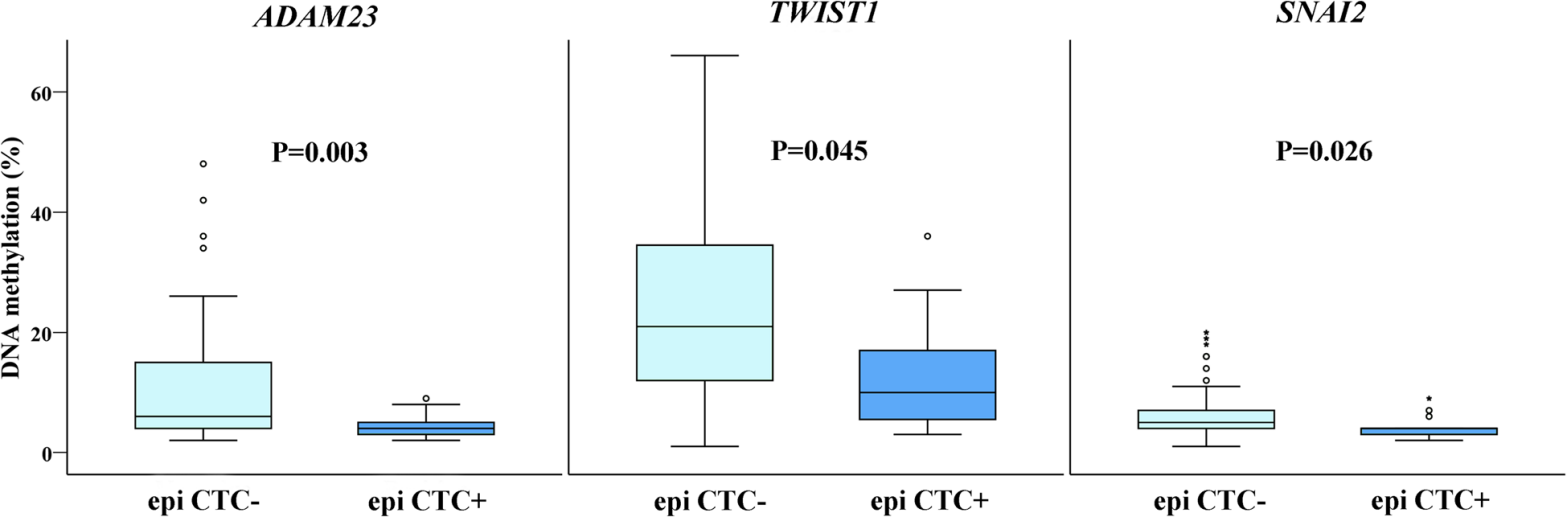
DNA methylation levels in tumours of patients without and with epithelial circulating tumour cells for *ADAM23, TWIST1* and *SNAI2* genes. The length of the boxes is the interquartile range (IQR) that represents values between the 75th and 25th percentiles. The circle (○) labelled outliers (values more than 1.5 IQRs but less than 3 IQRs from the end of the box). Values more than three IQRs, extremes, are depicted by asterisk (*).The horizontal line represents median. Significance level is regarded as *P* < 0.05. Abbreviations: epi CTC, epithelial circulating tumour cells

**Fig. 3:**
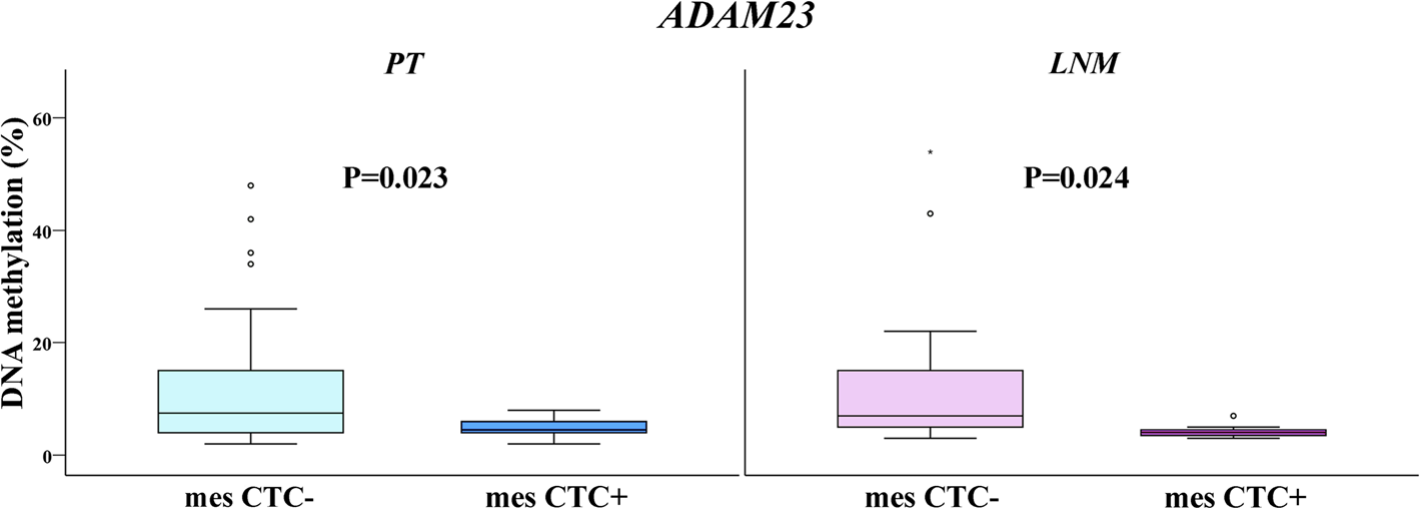
*ADAM23* methylation levels in PT and LMN samples of patients without and with mesenchymal circulating tumour cells. The length of the boxes is the interquartile range (IQR) that represents values between the 75th and 25th percentiles. The circle (○) labelled outliers (values more than 1.5 IQRs but less than 3 IQRs from the end of the box). Values more than three IQRs, extremes, are depicted by asterisk (*).The horizontal line represents median. Significance level is regarded as *P* < 0.05. Abbreviations: PT, primary tumour; LNM, lymph node metastases; mes CTC, mesenchymal circulating tumour cells

To evaluate the association between DNA methylation and haematogenous spread, we analysed DNA methylation levels in patients with epithelial CTCs (epi CTC+) and in those undergoing EMT process, as indicated by the presence of mesenchymal CTCs (mes CTC+) in their blood. We observed significantly lower methylation levels for *ADAM23, TWIST1* and *SNAI2* in the PTs of patients with epi CTC+ than in the PTs of patients with epi CTC- (4.45% vs. 11.46%, *P* = 0.003; 13.09% vs. 24.36%, *P* = 0.045 and 4.31% vs. 7.02%, *P* = 0.026, respectively) (Fig. 2). In BC patients with mes CTC+ in circulation, significant lower methylation levels of the *ADAM23* gene were identified in PT and LNM tissues than in the tissues of patients with mes CTC- (4.79% vs. 11.89%, *P* = 0.023 for PTs and 4.29% vs. 11.31%, *P* = 0.024 for LNMs) (Fig. 3). Additionally, we analysed methylation profiles in three groups of patients with epi CTC+, mes CTC+ and without any CTC (CTC-) and we found the significant differences in methylation levels in *SNAI1* gene between epi CTC+ and mes CTC+ patients, in *SNAI2* gene between epi CTC+ and CTC- patients. For *ADAM23* gene the similar values were observed between patients with epi CTC+ and mes CTC+, where DNA methylation was significantly lower for both these groups when compared to CTC- patients (Table 5).

**Table 5:** DNA methylation levels in primary tumours of CTC-negative and epithelial CTC and mesenchymal CTC-positive BC patients

	DNA methylation levels in primary tumour Mean ± std. deviation (in %)			
Genes	CTC-	epi CTC+	mes CTC+	*P* value*
*ADAM23*	14.17 ± 12.24^a,b^	4.56 ± 2.40^a^	4.92 ± 1.62^b^	**0.001**
*uPA*	13.29 ± 16.37	9.56 ± 8.75	19.33 ± 21.90	0.592
*CXCL12*	14.45 ± 14.09	7.10 ± 3.00	16.40 ± 15.51	0.287
*TWIST1*	24.08 ± 18.20	10.00 ± 8.55	24.86 ± 16.34	0.065
*SNAI1*	5.82 ± 7.14	2.89 ± 1.05^c^	8.40 ± 7.80^c^	**0.004**
*SNAI2*	7.11 ± 4.95^d^	3.70 ± 1.34^d^	6.85 ± 4.90	**0.022**

*Kruskal-Wallis Test; Adjusted pairwise comparisons: ^a^*P* = 0.003; ^b^*P* = 0.015; ^c^*P* = 0.004; ^d^*P* = 0.018; A statistical two-tailed significance was regarded as P value < 0.05. Abbreviations: *BC* breast cancer, *CTC* circulating tumour cell, *epi CTC* epithelial circulating tumour cells, *mes CTC* mesenchymal circulating tumour cells

**Fig. 4:**
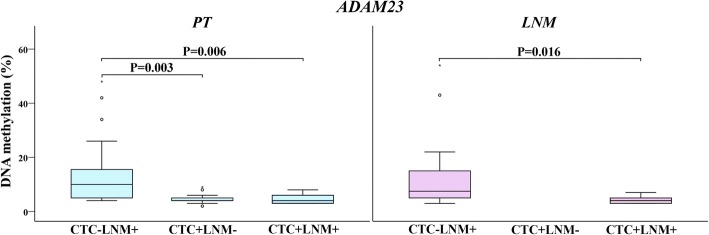
*ADAM23* methylation levels in PT and LMN samples of patients with different statuses of cancer cell dissemination characterized by CTC and/or LMN presence. The length of the boxes is the interquartile range (IQR) that represents values between the 75th and 25th percentiles. The circle (○) labelled outliers (values more than 1.5 IQRs but less than 3 IQRs from the end of the box). Values more than three IQRs, extremes, are depicted by asterisk (*).The horizontal line represents median. Significance level is regarded as *P* < 0.05. Abbreviations: PT, primary tumour; LNM, lymph node metastases; CTC, circulating tumour cells

We also evaluated DNA methylation in three groups of patients with different statuses of cancer cell dissemination, namely, CTC-negative patients with LNM (CTC-LNM+, *n* = 29), CTC-positive patients without LNM (CTC + LNM-, *n* = 16) and those positive for both parameters (CTC + LNM+, *n* = 13). For those analyses, one BC patient with micrometastasis in lymph node (LN) was excluded. We found significant differences in methylation levels among these three groups for *ADAM23* in PT and LNM tissues (Fig. 4) and *TWIST1* in PBCs.

The analysis of methylation levels in PTs and PBC samples between patients with exclusively haematogenous dissemination (CTC + LNM-) and those with only lymphogenous spread (CTC-LNM+) identified significant differences between methylation levels of the *ADAM23* gene in PTs and the *CXCL12* and *TWIST1* genes in PBC samples (4.69% vs. 13.39%, *P* = 0.002; 2.31% vs. 3.03%, *P* = 0.036; 3.31% vs. 4.48%, *P* = 0.008). Using multivariate logistic regression, we confirmed the inverse association between the *ADAM23* methylation level in PTs and the presence of CTCs in the peripheral blood of patients (OR = 0.45; 95% CI, 0.23–0.90; *P* = 0.023) (Table 6).

**Table 6:** The risk estimation of analysed variables and clinical status for exclusively haematogenous versus lymphogenous dissemination in breast cancer patients (logistic regression adjusted for age)

	Variables	*P* value	OR	95% CI
CTC + LNM- vs. CTC-LNM+	↑ Methylation of *ADAM23* in PT	0.023	0.45	0.229–0.898
	↑ Methylation of *CXCL12* in PBCs	0.046	0.07	0.005–0.949
	↑ Methylation of *TWIST1* in PBCs	0.050	0.03	0.001–0.998
	Tumour size > 20 mm	0.055	0.04	0.001–1.073

−2 Log likelihood = 15.22; *R*^2^(Cox and Snell) = 0.57; *R*^2^(Nagelkerke) = 0.81

*Abbreviations*: *LNM* lymph node metastases, *CTC* circulating tumour cells, *OR* odds ratio, *CI* confidence interval, ↑ continuous variable. Age and DNA methylation of genes significant in univariate analysis, hormone receptor and HER2 status, histological type, tumour size and Ki-67 proliferative index were analysed as independent variables, significant or borderline significant results are shown in the table

## Discussion

The high mortality rate associated with metastatic disease is a serious medical problem. To improve screening strategies and preventative treatment of metastatic progression in BC patients, detailed knowledge is needed about the molecular mechanisms that result in aggressive tumour phenotypes, including invasiveness and metastasis. In addition to many other molecular markers, DNA methylation biomarkers, particularly the specific alterations in methylation, offer tools for early detection, diagnosis and more effective management of advanced BC.

In the present study, we investigated promoter methylation in six genes associated with tumour invasivity (*ADAM23*, *uPA*, *CXCL12*, *TWIST1*, *SNAI1* and *SNAI2*). We found significantly different methylation levels for *CXCL12*, *TWIST1* and *SNAI2* genes in tumours of BC patients compared with healthy breast tissues, with mean values of 13.4%, 21.9% and 6.37%, respectively; however, these relations could be influenced by the fact that BC patients were older than healthy donors. With the process of aging, especially after the menopause, mammary glands undergo involution. Both glandular parenchyma and connective tissue atrophy with reduction of adipose tissue. These morphological changes are accompanied with molecular alterations including DNA methylation. In normal breast tissues, the age-related methylation in 204 CpG loci was observed, which progressively alterated in breast tumours [58]. Therefore, for evaluation of cancer-specific methylation profiles, the younger persons cannot be accepted as accurate controls to BC patients, who undergone the aging processes.

In 206 patients, the methylation level of the *CXCL12* gene was 12.04% [17]; however, in another laboratory, quantitative methylation analyses of *CXCL12* gene were performed in only three BC samples, with variable methylation levels detected, ranging from 6 to 89% [30]. Other authors observed slightly lower mean levels of *TWIST1* methylation than we evidenced in our study, namely, 17.5% and 15.3% [35, 36], but Gort and colleagues determined a higher methylation level of *TWIST1* in 76 samples of invasive BC, with a mean of 34% [37]. The median of our results for *TWIST1* methylation (18.5%) is approximately two-fold higher than the median of 8.85% measured in BC patients by other researchers using the same pyrosequencing method [59]. In BC cell lines, an inverse association between DNA methylation and the transcription levels of the *SNAI1* and *SNAI2* genes has been documented [40]. These in vitro results together with ours in BC patients indicate that in addition to *CXCL12* and *TWIST1* methylation, the aberrant methylation profile of the *SNAI2* gene could also contribute to the invasivity of BC.

For the appropriate staging of BC, the tumour size and lymph node status are considered to be the most important clinical categories. However, the histologic grade, which characterizes the degree of cancer cell differentiation, has not yet been integrated into the TNM classification. In a recent meta-analysis, the prognostic importance of histologic grade for overall survival was documented, without any association with tumour size or nodal status [60]. Moreover, integrative bioinformatics analyses have identified the 22-gene tumour aggressiveness grading classifier that indicate the variable features of cancer cells in tumour tissues with different levels of de-differentiation because of individual sets of aberrant genetic changes [61]. To the best of our knowledge, no study has been published that investigated the association between changes in whole-genome methylation profiles and cancer de-differentiation processes.

It is generally accepted that the SLUG (SNAI2) transcription factor, encoded by the *SNAI2* gene, induces EMT, initiating cancer dissemination processes. This fact was supported by a previous study, in which the higher expression levels of the SLUG protein and *SLUG* mRNA were observed in the tumours of patients with metastatic BC or disease recurrence [34]. Moreover, the results of an in vitro study documented epigenetic regulation through promoter methylation in both the *SNAI1* and *SNAI2* genes during EMT and reverse mesenchymal-epithelial transition [40]. However, our results indicate the other possible role of the *SNAI2* gene in BC, which is the de-differentiation of cancer cells. *SNAI2* involvement in cell differentiation has been shown in human epidermal progenitor cells. Overexpressed *SNAI2* inhibits the differentiation of these cells by binding to the differentiation and adhesion genes across the genome. On the other hand, low levels of *SNAI2*, and therefore weak binding during epidermal differentiation, lead to the full expression of the differentiation programme. Similar processes could be expected in cancer cells in which high levels of *SNAI2* result in more intensive binding of SNAI2 to the genomic targets, determining the differentiation status of epithelial cells. Cancer cells then trigger EMT and the inhibition of cell differentiation [62]. Based on the assumption that decreased methylation could allow the up-regulation of *SNAI2* gene expression, reduced differentiation in tumours could be expected, as was observed in our study by the inverse relationship between *SNAI2* methylation and histologic grade.

Two important roles in cancer cell spread are played by ADAM proteins performing proteinase activities through the metalloproteinase domain and regulating cell adhesion by their interaction with integrins, which participate in tumour growth and metastasis as well as in tumour angiogenesis [63]. Among the ADAM proteins, proteolytically inactive ADAM23 negatively regulates cancer cell migration by binding αvβ3 integrin to its disintegrin domain [15]. In addition to cell migration, the active integrins promote the interaction between CTCs and thrombocytes, thereby mediating the protection of cancer cells against degradation in the vasculature [64]. In BC cell lines, Costa and colleagues found that *ADAM23* promoter hypermethylation was strongly associated with the reduction in mRNA and protein expression, and they also observed higher methylation levels in PTs with more advanced grade [16]. In our study, we investigated whether there are differences in methylation levels in BC patients with different routes of cancer dissemination. We found significantly lower *ADAM23* methylation levels in tumours of CTC-positive patients, regardless of their epithelial or mesenchymal phenotype, than in tumours of patients with LNM. Our results indicate that the higher expression level of the *ADAM23* gene could contribute to its newly identified function in the haematogenous dissemination of BC.

The dissemination of cancer cells is performed via blood and lymphatic vessels after neo-vascularization and neo-lymphangiogenesis in tumour tissue [65]. In a very interesting study, the molecular features of endothelial cells (EC) located in tumour-associated vasculature were studied to evaluate a new strategy to inhibit tumour growth and cancer dissemination. In this model of EC purified and cultured from ovarian cancer and normal adrenal glands as control tissues, the authors identified 158 highly expressed transcripts. Of these mRNAs, the *ADAM23*, *FAP*, *GPMNB* and *PRSS3* were found in the tumour-derived endothelium, but no expression was observed in tumour cells. Moreover, *ADAM23*, *GPMNB* and *PRSS3* expression only occurred in the blood vessels of human cancer samples [66]. These findings seem to not be organ-specific but rather to be exclusive to tumour-derived EC because expression of several genes that are typical for tumour vasculature was also observed in other types of malignancies in the colon, brain and breast [67–69]. In the context of our results, we consider that decreased methylation of the *ADAM23* gene could partially represent the molecular profile of EC from the tissue surrounding the tumour body rather than from the tumour cells. Therefore, patients with higher *ADAM23* expression levels, which may be the result of decreased promoter methylation, could have contributed to the dense development of tumour blood vessels, resulting in the haematogenous rather than the lymphogenous spread of cancer cells.

## Conclusions

In our study, among the selected genes related to the partial processes of cancer cell invasivity, we identified higher cancer-specific methylation levels in the *CXCL12*, *TWIST1* and *SNAI2* genes. In contrast to *CXCL12* and *TWIST1* methylation, the epigenetic regulation of the *SNAI2* gene was previously determined only in BC cell line models, not in tumour samples. Furthermore, the decreased methylation of the *SNAI2* gene in tumours with histologic grade 3 indicates a new function of a typical EMT gene in cancer cell de-differentiation. The differences in *ADAM23* methylation profiles in BC patients with various route of cancer cells dissemination suggest that ADAM23 could participate in haematogenous spread. Our research uncovers new relationships between aberrant methylation profiles and clinical characteristics of advanced BC. However, experimental investigation of the associated molecular mechanisms is needed before the *SNAI2* and *ADAM23* methylation profiles can contribute to the management of metastatic progression in BC.

## Additional file

Additional file 1: DNA methylation levels in various types of samples from BC patients with different histological grade. A table containing methylation results for all six genes and statistical evaluation between two groups of patients with G1 + G2 and G3 tumours in samples of peripheral blood cells, primary tumours and lymph node metastases. (DOCX 15 kb)

## Abbreviations

ADAM: A disintegrin and metalloproteases
ANOVA: Analysis of variance
BC: Breast cancer
BM: Basement membrane
CD45+: CD45-positive
CTCs: Circulating tumour cells
EC: Endothelial cell
ECM: Extracellular matrix
EMT: Epithelial-mesenchymal transition
epi CTC: Epithelial circulating tumour cell
FFPE: Formalin fixed paraffin-embedded tissue
G: Grade
HGF: Hepatocyte growth factor
HR: Hormonal receptor
LN: Lymph node
LNMs: Lymph node metastases
mes CTC: Mesenchymal circulating tumour cell
PBCs: Peripheral blood cells
PT: Primary tumour
qRT-PCR: Quantitative real-time polymerase chain reaction
TFs: Transcription factors
TN: Triple-negative
VEGF: Vascular endothelial growth factor
