# Supplementary material for: Decreased methylation in the SNAI2 and ADAM23 genes associated with de-differentiation and haematogenous dissemination in breast cancers
Source: BMC Cancer. 2018 Sep 6;18:875. doi: 10.1186/s12885-018-4783-x (PMC6127923; doi:10.1186/s12885-018-4783-x)
Supplement: Supplementary file 1 — DNA methylation levels in various types of samples from BC patients with different histological grade. A table containing methylation results for all six genes and statistical evaluation between two groups of patients with G1 + G2 and G3 tumours in samples of peripheral blood cells, primary tumours and lymph node metastases. (DOCX 15 kb) [file 12885_2018_4783_MOESM1_ESM.docx]

|  | **DNA methylation levels in peripheral blood cells**  **Mean ± std. deviation (in %)** | |  | **DNA methylation levels in primary tumour**  **Mean ± std. deviation (in %)** | |  | **DNA methylation levels in lymph node metastases**  **Mean ± std. deviation (in %)** | |  |
| --- | --- | --- | --- | --- | --- | --- | --- | --- | --- |
| **Genes** | **G1+G2** | **G3** | ***P* value** | **G1+G2** | **G3** | ***P* value** | **G1+G2** | **G3** | ***P* value** |
| *ADAM23* | 2.39 ± 0.56 | 2.65 ± 0.75 | 0.222 | 9.90 ± 10.39 | 10.45 ± 10.60 | 0.808 | 9.29 ± 9.20 | 10.87 ± 13.20 | 0.808 |
| *uPA* | 2.15 ± 0.76 | 2.35 ± 0.93 | 0.653 | 10.28 ± 10.52 | 21.65 ± 23.98 | 0.078 | 9.25 ± 13.46 | 9.89 ± 10.72 | 0.901 |
| *CXCL12* | 3.06 ± 1.32 | 2.35 ± 0.59 | **0.021** | 10.89 ± 9.78 | 17.48 ± 17.72 | 0.128 | 10.14 ± 7.74 | 13.60 ± 12.83 | 0.276 |
| *TWIST1* | 4.12 ± 1.43 | 4.25 ± 1.45 | 0.753 | 22.06 ± 16.79 | 21.72 ± 17.44 | 0.868 | 12.45 ± 11.15 | 20.80 ± 14.90 | 0.134 |
| *SNAI1* | 2.12 ± 0.96 | 2.25 ± 1.12 | 0.658 | 4.71 ± 3.20 | 8.26 ± 10.26 | 0.387 | 5.39 ± 1.72 | 3.42 ± 1.98 | **0.007** |
| *SNAI2* | 3.48 ± 0.76 | 3.50 ± 0.89 | 0.992 | 7.38 ± 4.77 | 4.00 ± 1.41 | **0.003** | 6.83 ± 6.66 | 2.67 ± 1.23 | 0.062 |

**Additional file 1 DNA methylation levels in various types of samples from BC patients with different histological grade**

Abbreviations: BC, breast cancer; G, grade.
